# Beer, Cider, and Wine Allergy

**DOI:** 10.1155/2017/7958924

**Published:** 2017-03-15

**Authors:** Rhea A. Bansal, Susan Tadros, Amolak S. Bansal

**Affiliations:** ^1^Immunology Department, St Helier Hospital, Wrythe Lane, Carshalton, Surrey SM5 1AA, UK; ^2^St Helier Hospital, Wrythe Lane, Carshalton, Surrey SM5 1AA, UK

## Abstract

*Background*. Allergy to beer is often due to specific proteins in barley and sometimes to lipid transfer protein. Allergy to wine is frequently due to a sensitivity to grape proteins. We present a rare case of allergy to beer, wine, and cider resulting from IgE reactivity to yeasts and moulds which also explained the patient's additional sensitivity to yeast extracts and blue cheese.* Case Presentation*. The patient's symptoms included throat and facial itching accompanied by mild wheeze and severe urticaria. Diagnosis of allergy to yeast was confirmed by specific IgE testing as well as that to relevant foods and beverages. The patient's ongoing management included advice to avoid beer, wine, and other food groups containing specific yeasts, in addition to carrying a short acting nonsedating antihistamine as well as an adrenaline autoinjector.* Conclusions*. Cases of yeast allergy are extremely rare in medical literature but may be underrecognised and should be considered in patients presenting with reactions to alcoholic beverages and other yeast-containing products.

## 1. Introduction

Medically confirmed IgE-mediated yeast allergy is exceptionally rare. Our case provides a clear history of immediate hypersensitivity to yeasts and yeast components with symptoms being evident shortly after ingestion of several yeast-containing alcoholic beverages.

In addition, we highlight the usefulness of fresh food skin prick testing as an easy, inexpensive, and rapid technique to check for immediate IgE-mediated reactivity in patients with allergic-type symptoms. Interestingly, the previous medical literature on allergic reactions to wine and beer has been attributed to grape and lipid transfer proteins, with almost no mention of allergy to yeast or yeast components. As such, our case represents a very rare and possibly unique condition.

## 2. Case Presentation

A 19-year-old young man described episodes of itching of his throat within a minute of ingesting beer, cider, wine, champagne, and marmite over the previous several years. On some occasions, this was accompanied by mild wheezing and facial itching. With craft/microbrewery beer (often made with locally sourced ingredients), champagne, and wine, the reactions were more severe with constriction of the throat, wheezing, nasal blockage, and severe urticaria. Each of these reactions lasted for approximately one hour and was ameliorated with oral antihistamines and occasionally a salbutamol inhaler was also required. Such reactions occurred on every occasion that the patient drank wine or beer products. He had no problems consuming spirits such as gin and vodka. In addition, this patient also described two episodes of itching of his mouth and throat after eating a banana. He tolerated apples, grapes, avocados, and several of the common draft beers and lagers without symptoms. Of interest, the patient had never experienced symptoms after eating yeast-containing baked products such as bread and pizza. He suffered from seasonal allergic rhinitis and asthma and had a past history of childhood eczema. There was no other relevant past medical history. He denied problems taking nonsteroidal anti-inflammatory agents and was not on any regular medications. There was no significant mould or dampness in his home or work and he had no exposure to pets.

## 3. Investigations and Diagnosis

The patient's routine blood tests confirmed a normal white cell count of 5.95 × 10^6^/L without eosinophilia. Complement proteins C3 and C4 were both normal and there was no evidence of autoimmunity to nuclear, liver, and stomach antigens. The tryptase was also normal at 3.9. The total IgE was 95 kU/L and the specific IgE to yeast was elevated at 2.32 kUA/L. The specific IgE to a mould mix containing penicillium, cladosporium, aspergillus fumigatus, and alternaria was also high at 7.09 kUA/L and that to* Candida* was raised at 2.24 kUA/L (ImmunoCap, Thermo Fisher Scientific Inc., Uppsala, Sweden).  Table 1 below shows the results of skin prick testing. Testing of the food and alcohol products was performed by using a prick-to-prick technique.

## 4. Treatment and Outcomes

Our patient's skin and blood results confirmed yeast and mould sensitivity as the cause of his reactivity to yeast extracts and alcoholic beverages made with yeast.

He was advised to avoid consuming beers, ciders, and wine, as well as vegemite, marmite, and other foods containing yeast extract. He was prescribed antihistamines and advised to carry an adrenaline autoinjector. This advice was particularly important as he was shortly due to travel for several months to remote destinations. However, his previous reactions were not associated with any significant cardiorespiratory compromise.

## 5. Discussion

There is scarce literature on allergy to yeasts, although IgG anti-*Saccharomyces cerevisiae* autoantibodies have been implicated in the development of Crohn's disease and have also been detected in various autoimmune conditions such as antiphospholipid syndrome, SLE, type 1 diabetes, and rheumatoid arthritis [1]. However, Pajno et al. described a young boy who developed wheezing and generalised urticaria after eating freshly prepared pizza and bread. Sensitisation was confirmed specifically to baker's yeast, and it was interesting that the boy did not experience any symptoms when eating yeast-containing baked foods prepared over an hour earlier. It was suggested that the* Saccharomyces* spp. may lead to more significant allergic reactions when airborne and inhaled, rather than when ingested [2].

Beer is one of the world's most commonly consumed beverages and ranks just below water and tea. It is produced by the fermentation of malted barley but can also be made from other starches such as corn, wheat, maize, rice, millet,* Sorghum*, cassava, and* Agave*. The staple ingredients contained in beer include water, starch, brewer's yeast, and hops for flavouring. The main types of yeast used in the fermentation process are* Saccharomyces cerevisiae* and the highly related* Saccharomyces pastorianus* as well as* Brettanomyces* spp. and* Torulaspora delbrueckii*. The precise yeast depends on what type of beer that is being produced, that is, ale or lager and also where in the world it is brewed [3]. In contrast, wines and ciders utilise natural yeasts and vegemite and marmite are yeast extracts from* S. cerevisiae*.

In our patient, the largest skin test wheals were evident with the yeast extracts. This was likely related to the higher concentrations of yeast proteins here. Boiling the beer did not reduce the skin test reactivity. This is unsurprising as most commercial canned beers are heated to kill the yeast and stop further fermentation and our patient reacted to both bottled and canned beers. The variation on skin test reactivity with the different beers may be due to the different yeasts used. Thus the lagers using top fermentation utilise* S. pastorianus*, while others use a bottom fermentation and* S. cerevisiae*. Interestingly, our patient had significant specific IgE and skin reactivity not only to the common culinary yeasts but also to* Candida* and other fungi. His sensitivity to wine and cider was likely due to the contained yeasts as he tolerated grapes [4] and apple without reactivity. It is possible that the variable tolerance of our patient to the different beers may perhaps be due to the different strains of yeast used in the production.

The worldwide differences in the ingredients and methods used for brewing beer create a real challenge in the identification and characterisation of culprit allergens in cases of suspected beer allergy. For example, a case study of 27 individuals of Chinese origin who experienced symptoms of allergic sensitivity after drinking beer (most commonly urticaria and erythema of the skin) showed that one-third of these had positive skin prick tests to* Sorghum*, the main fermented ingredient in common Chinese alcoholic beverages [5].

Much of the literature surrounding allergy to beer attributes this to a sensitivity to barley extracts. A 10 kD barley protein was previously identified as the factor responsible for urticaria caused by beer consumption [6]. Similarly, in 2 cases of anaphylaxis following beer ingestion reported by Bonadonna et al. [7], serum-specific IgE testing revealed barley as the responsible ingredient. Later literature identified both a 9 kD lipid transfer protein 1 (LTP1) and the 45 kD Z4 barley protein as the major allergens in beer [8]. Interestingly, Asero et al. noted that the LTP causing beer allergy in their patient strongly cross-reacted with LTPs from peach, carrot, and apple [9]. In our case, skin prick testing was negative for the 7 Cereal Mix (containing barley), thus making this an unlikely cause of his allergy. Furthermore, he also reacted to nonbarley containing beverages including wine and cider.

Other causes of reactivity to alcohol include a very unusual “allergy” to acetic acid, as stated by Nakagawa et al. [10]. Here a middle-aged man experienced generalised urticaria and lip swelling for a period of 2 years after drinking various types of alcoholic beverages. While skin prick testing with beer, shochu, brandy, ethanol, and acetaldehyde was negative, it was inexplicably positive to acetic acid. A similar case described more than 2 decades previously involved a young woman who developed generalised itching, facial flushing, dyspnoea, and collapse after ingesting wine, beer, rum, and vinegar. Following skin prick testing, the cause of her allergic symptoms was also attributed to acetic acid [11]. How acetic acid, an extremely small molecule, can undergo specific binding to IgE is unclear. In regard to wine allergy, Sbornik et al. [12] described an elderly man who developed facial swelling and respiratory distress on two occasions, firstly after drinking red wine and subsequently after eating grapes. They noted specific IgE reactivity to 3 proteins in grape and red wine extracts by Western Blot analysis; 30-kD-endochitinase 4A and 4B, 24-kD-thaumatin-like protein, and 9-kD-lipid transfer protein. Clinically, this patient differs from ours in that he could drink beer which ours could not and our patient tolerated grapes whereas he could not. Interestingly lipid transfer proteins appear to be one of the more important allergenic components in grape and have high a degree of homology to those in peaches and cherry [13, 14]. This may account for at least some of the allergic cross-reactivity between these fruits.

In conclusion, we describe an unusual case of IgE-mediated mould and yeast sensitivity contributing to localised and systemic reactions with beer, wine, and cider. Such allergens present significant challenges in the diagnosis and ongoing management of such patients due to their widespread prevalence in food and beverages. Further work is required to establish the prevalence of yeast sensitivity as well as identification of the specific yeast proteins that cause these allergic reactions.

## Figures and Tables

**Table 1:** Skin prick and raw food testing results.

Alcohols, yeasts, and moulds	Wheal size (mm)	Foods	Wheal size (mm)
Cider	8	Blue cheese	6
Red wine	5	Vegemite	18
Indian pale ale	9 (boiled)	7 Cereal Mix	0
	6 (fresh)	Corn	0
Italian pale lager	4	Wheat	2
Brewer's yeast	12		
Mould mix I	8	Cantaloupe melon	5
Mould mix II	10	Banana	0
*Candida*	4-5		
*Aspergillus fumigatus *	3-4		
